# Increased risk of Parkinson’s disease following tension-type headache: a nationwide population-based cohort study

**DOI:** 10.18632/oncotarget.23298

**Published:** 2017-12-14

**Authors:** Fu-Chi Yang, Hsuan-Ju Chen, Jiunn-Tay Lee, Sy-Jou Chen, Yueh-Feng Sung, Chia-Hung Kao, Tse-Yen Yang

**Affiliations:** ^1^ Department of Neurology, Tri-Service General Hospital, National Defense Medical Center, Taipei, Taiwan; ^2^ Management Office for Health Data, China Medical University Hospital, Taichung, Taiwan; ^3^ Department of Emergency Medicine, Tri-Service General Hospital, National Defense Medical Center, Taipei, Taiwan; ^4^ Graduate Institute of Injury Prevention and Control, College of Public Health and Nutrition, Taipei Medical University, Taipei, Taiwan; ^5^ Graduate Institute of Clinical Medical Science and School of Medicine, College of Medicine, China Medical University, Taichung, Taiwan; ^6^ Department of Nuclear Medicine and PET Center, China Medical University Hospital, Taichung, Taiwan; ^7^ Department of Bioinformatics and Medical Engineering, Asia University, Taichung, Taiwan; ^8^ Department of Medical Laboratory Science and Biotechnology, China Medical University, Taichung, Taiwan; ^9^ Department of Medical Research, China Medical University Hospital, Taichung, Taiwan; ^10^ Molecular and Genomic Epidemiology Center, China Medical University Hospital, Taichung, Taiwan

**Keywords:** tension-type headache, Parkinson’s disease, retrospective cohort study, National Health Insurance Research Database

## Abstract

**Purpose:**

Previous studies have suggested associations between primary headache and neurodegenerative diseases; however, the relationship between tension-type headache (TTH), which is the most common type of primary headache, and Parkinson's disease (PD) remains controversial. Hence, in this nationwide, population-based, retrospective cohort study, we explored the temporal association between TTH and PD.

**Methods:**

Using claims data in the National Health Insurance Research Database of Taiwan, we evaluated 12,309 subjects aged ≥20 years who were newly diagnosed with TTH from 2000 to 2005. The non-TTH group included 49,236 randomly selected sex- and age-matched patients without TTH. Subjects were followed up until the end of 2011, diagnosis of PD, or death. The incidence of PD was compared between the two groups. A Cox multivariable proportional hazards model was used to calculate hazard ratios (HRs) and 95% confidence intervals (CIs) to estimate the risk of PD.

**Results:**

The overall incidence of PD (per 1,000 person-years) in the TTH and non-TTH groups was 3.01 and 1.68, respectively. After adjustment for sex, age, and comorbidities, the association between TTH and PD remained statistically significant (adjusted HR = 1.37, 95% CI = 1.19–1.57). The TTH group had a higher risk of PD than the non-TTH group did, regardless of subjects’ sex, age, and comorbidity status.

**Conclusions:**

These findings demonstrate that patients diagnosed with TTH exhibit an increased risk of PD. Additional studies should investigate the potential shared pathophysiological mechanisms of TTH and PD. Clinicians should be aware that TTH is a potential risk factor for PD.

## INTRODUCTION

Parkinson's disease (PD) is a progressive neurodegenerative disorder that affects more than 1% of the elderly population worldwide [1]. PD is characterized by motor-related symptoms including bradykinesia, rigidity, and resting tremor, which are caused by the progressive loss of nigrostriatal dopaminergic neurons [2]. The risk for PD increases with age, although other risk factors have been reported. These include various medical disorders or comorbidities, such as diabetes, hyperlipidemia, hypertension, ischemic heart disease, dementia, depression, migraine, stroke, chronic kidney disease, and head injury [2–6]. Although PD is predominantly regarded as a movement disorder, accumulating evidence indicates that PD patients also experience a wide range of nonmotor symptoms [7]. Nonmotor symptoms, including cognitive, psychiatric, sleep, autonomic, sensory, and pain disorders, can develop before the onset of motor symptoms [8]. Pain is one of the most frequent nonmotor symptoms reported by PD patients [9]. However, the mechanisms of pathological pain in PD are poorly understood.

Headache is a pain symptom that is often associated with neurological disorders, and tension-type headache (TTH) is the most common type of primary headache [10]. TTH patients typically present with bilateral, nonthrobbing headache of mild-to-moderate intensity, which is usually caused by mental tension or stress [11]. TTH accounts for 46% of the global prevalence of headache compared with 14% for migraines [12]. Although TTH is generally less severe than migraines, it has a substantial socioeconomic impact on the general population.

Previous studies have suggested a relationship between primary headache and neurodegenerative diseases such as dementia and cognitive decline [13–16]. However, the association between primary headache and PD has been studied less and remains controversial. Barbanti et al. identified a lifetime migraine prevalence of 27.8% and a current migraine prevalence of 13.1% in a population of PD patients [17]. Furthermore, a cohort study showed that subjects with a midlife history of headache and particularly those with migraine with aura had an increased likelihood of movement disorders or PD [6]. In contrast to these studies, Lorentz et al. identified no significant differences in the total prevalence of headache among PD patients in a controlled study [18]. Furthermore, a later study demonstrated that PD patients had a lower lifetime prevalence of headache than control subjects did [19]. Previous studies have focused on the relationship between migraine and PD, and a prior validation study reported that 80% of individuals with non-migrainous headache might have TTH [20]. However, the relationship between TTH, which is the most prevalent type of primary headache, and PD is still unknown worldwide and in Asia.

Based on the collective evidence, the association between primary headache and PD remains controversial, and the temporal relationship between TTH and PD is still unknown. Therefore, in this nationwide, population-based cohort study, we aimed to explore the possible temporal association between TTH and PD. We tested the hypothesis that patients with TTH exhibit an increased risk for subsequent PD than patients without TTH after adjustment for potential confounding factors.

## RESULTS

In this study, the TTH group comprised 12,309 patients with newly diagnosed TTH, and the non-TTH group comprised 49,236 subjects without TTH who were frequency matched by sex, age, and index-year (Table 1). The distributions of sex and age were the same in the two groups. The mean age of the TTH patients was 49.1 (SD = 15.4) years, with a slight predominance of women (approximately 66.8%). Compared with the non-TTH group, the TTH group had a higher prevalence of comorbidities, including diabetes, hyperlipidemia, hypertension, IHD, depression, migraine, stroke, and head injury (*P* <.001).

**Table 1:** Baseline demographic factors and comorbidity of study participants according to tension-type headache status

Characteristics	Non-TTH group N = 49236		TTH group N = 12309		p-value
	n	%	n	%	
Sex					> 0.99
Women	32872	66.8	8218	66.8	
Men	16364	33.2	4091	33.2	
Age, years					> 0.99
20-44	20976	42.6	5244	42.6	
45-64	19372	39.4	4843	39.4	
≥ 65	8888	18.1	2222	18.1	
Mean (SD)^†^	48.8	(15.6)	49.1	(15.4)	0.13
Comorbidity					
Diabetes	4243	8.62	1265	10.3	<0.001
Hyperlipidemia	8039	16.3	3206	26.1	<0.001
Hypertension	12235	24.9	4560	37.1	<0.001
IHD	6523	13.3	2803	22.8	<0.001
Dementia	356	0.72	98	0.80	0.43
Depression	1613	3.28	1362	11.1	<0.001
Migraine	954	1.94	1341	10.9	<0.001
Stroke	1075	2.18	338	2.75	<0.001
CKD	583	1.18	161	1.31	0.28
Head injury	1957	3.97	978	7.95	<0.001

TTH, tension-type headache; SD, standard deviation; IHD, ischemic heart disease; CKD, chronic kidney disease.

^†^ Student's t-test.

**Figure 1:**
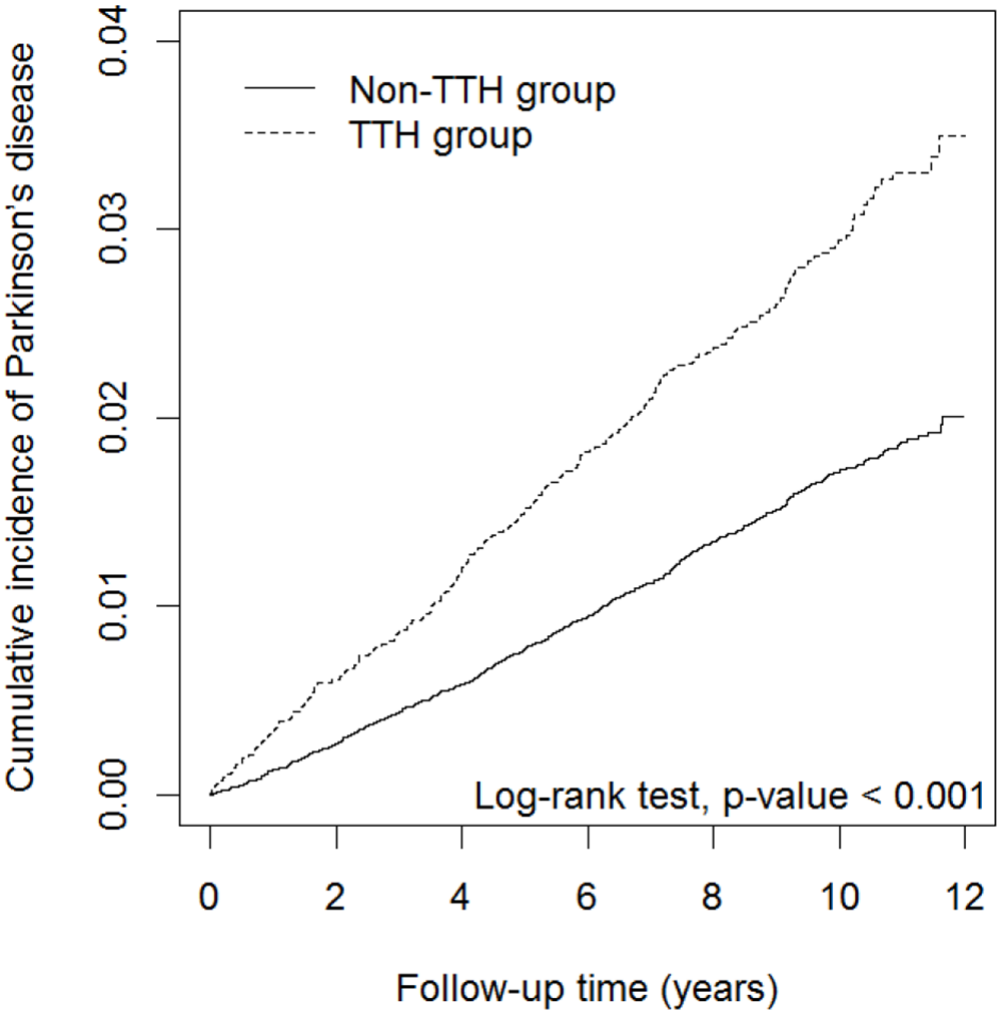
Cumulative incidence curves of Parkinson's disease for groups with (TTH) and without tension-type headache (non-TTH)

Figure 1 displays the cumulative incidence curves of PD for the non-TTH and TTH groups. We observed that the cumulative incidence of PD was significantly higher in patients with TTH than in subjects without TTH (log-rank test, *P* <.001). The mean follow-up period for this study population (TTH and non-TTH groups) was 8.55 years. The mean follow-up period for the TTH and non-TTH groups were 8.63 (SD = 2.36) years and 8.53 (SD = 2.42) years, respectively. The number of patients diagnosed with PD was 320 in the TTH group, with an incidence density rate of 3.01 per 1,000 person-years, and 708 patients in the non-TTH group, with an incidence density rate of 1.68 per 1,000 person-years. Within the TTH group, 320 patients were newly diagnosed with PD after a mean follow-up (SD) of 4.61 (2.92) years at a mean age (SD) of 68.4 (13.6). Within the non-TTH group, 708 patients were newly diagnosed with PD after a mean follow-up (SD) of 4.91 (2.81) years at a mean age (SD) of 72.8 (10.3) years. After adjustment for sex, age, and comorbidities, the TTH group had a significantly higher risk of PD than did the non-TTH group (adjusted HR = 1.37, 95% CI = 1.19–1.57; Table 2). Compared with the 20–44 age group, the risk of PD was 4.66-fold (95% CI = 3.46–6.26) and 20.4-fold (95% CI = 15.2–27.5) higher in the 45–64 and ≥65 age groups, respectively. This result indicated that the risk of PD increased with age. The multivariable Cox proportion hazards regression model revealed that patients with diabetes, hypertension, IHD, dementia, depression, and migraine had a higher risk of PD than their counterparts did (adjusted HR = 1.27, 95% CI = 1.09–1.48; adjusted HR = 1.55, 95% CI = 1.32–1.80; adjusted HR = 1.32, 95% CI = 1.15–1.52; adjusted HR = 2.25, 95% CI = 1.66–3.04; adjusted HR = 2.19, 95% CI = 1.82–2.63; and adjusted HR = 1.46, 95% CI = 1.14–1.86, respectively).

**Table 2:** Cox model measured hazard ratios and 95% confidence interval of Parkinson's disease associated with tension-type headache and covariates

Characteristics	Event no.	IR	HR (95% CI)	
			Univariate	Multivariate^†^
Tension-type headache				
No	708	1.68	1.00	1.00
Yes	320	3.01	1.79 (1.57-2.04)^***^	1.37 (1.19-1.57)^***^
Sex				
Women	653	1.83	1.00	1.00
Men	375	2.22	1.22 (1.07-1.38)^**^	1.05 (0.92-1.19)
Age, years				
20-44	54	0.23	1.00	1.00
45-64	304	1.43	6.19 (4.63-8.27)^***^	4.66 (3.46-6.26)^***^
≥ 65	670	8.17	35.8 (27.1-47.2)^***^	20.4 (15.2-27.5)^***^
Comorbidity				
Diabetes				
No	795	1.64	1.00	1.00
Yes	233	5.50	3.37 (2.91-3.90)^***^	1.27 (1.09-1.48)^**^
Hyperlipidemia				
No	625	1.44	1.00	1.00
Yes	403	4.34	3.02 (2.66-3.42)^***^	1.14 (0.99-1.30)
Hypertension				
No	345	0.88	1.00	1.00
Yes	683	5.08	5.81 (5.11-6.61)^***^	1.55 (1.32-1.80)^***^
IHD				
No	579	1.28	1.00	1.00
Yes	449	6.11	4.81 (4.25-5.44)^***^	1.32 (1.15-1.52)^***^
Dementia				
No	981	1.87	1.00	1.00
Yes	47	18.5	10.2 (7.59-13.6)^***^	2.25 (1.66-3.04)^***^
Depression				
No	878	1.75	1.00	1.00
Yes	150	6.35	3.66 (3.07-4.35)^***^	2.19 (1.82-2.63)^***^
Migraine				
No	952	1.88	1.00	1.00
Yes	76	4.02	2.15 (1.71-2.72)^***^	1.46 (1.14-1.86)^**^
Stroke				
No	956	1.85	1.00	1.00
Yes	72	8.10	4.46 (3.51-5.67)^***^	1.07 (0.83-1.37)
CKD				
No	990	1.90	1.00	1.00
Yes	38	8.03	4.30 (3.11-5.95)^***^	1.36 (0.98-1.89)
Head injury				
No	949	1.89	1.00	1.00
Yes	79	3.39	1.80 (1.43-2.27)^***^	1.20 (0.95-1.51)

IR, incidence density rates, per 1000 person-years; HR, hazard ratio; CI, confidence interval; IHD, ischemic heart disease; CKD, chronic kidney disease.

^†^ Multivariate Cox proportional hazards regression model including tension-type headache, sex, age, diabetes, hyperlipidemia, hypertension, IHD, dementia, depression, migraine, stroke, CKD, and head injury.

^**^ *P*<.01, ^***^ *P*<.001.

After stratification by sex, we determined that the women and men in the TTH group had a higher risk of PD than did those in the non-TTH group (adjusted HR = 1.41, 95% CI = 1.18–1.67 and adjusted HR = 1.31, 95% CI = 1.04–1.66, respectively). After stratification by age group, we concluded that the TTH group had a higher risk of PD than the non-TTH group did for all age groups. The adjusted HRs of PD were 2.71 (95% CI = 1.48–4.97) for the 20–44 age group, 1.45 (95% CI = 1.13–1.87) for the 45–64 age group, and 1.24 (95% CI = 1.04–1.48) for the ≥65 age group. After stratification by comorbidity status, we determined that the patients with and without comorbidities in the TTH group had a higher risk of PD than did their counterparts in the non-TTH group (adjusted HR = 1.56, 95% CI = 1.35–1.80 and adjusted HR = 1.54, 95% CI = 1.03–2.31, respectively; Table 3).

**Table 3:** Incidence density rates and hazard ratios of Parkinson's disease for the effect of tension-type headache stratified by sex, age, and comorbidity

Characteristics	Non-TTH group			TTH group			Compared to the non-TTH group	
							HR (95% CI)	
	Event no.	Person-years	IR	Event no.	Person-years	IR	Crude	Adjusted^‡^
Sex								
Women	449	285389	1.57	204	72083	2.83	1.80 (1.52-2.12)^***^	1.41 (1.18-1.67)^***^
Men	259	134817	1.92	116	34099	3.40	1.77 (1.42-2.21)^***^	1.31 (1.04-1.66)^*^
Age, years								
20-44	24	185474	0.13	30	47031	0.64	4.91 (2.87-8.40)^***^	2.71 (1.48-4.97)^**^
45-64	201	169427	1.19	103	42482	2.42	2.04 (1.61-2.59)^***^	1.45 (1.13-1.87)^**^
≥ 65	483	65304	7.40	187	16670	11.2	1.52 (1.28-1.79)^***^	1.24 (1.04-1.48)^*^
Comorbidity status^†^								
No	144	263567	0.55	28	42659	0.66	1.19 (0.80-1.79)	1.54 (1.03-2.31)^*^
Yes	564	156639	3.60	292	63523	4.60	1.28 (1.11-1.47)^***^	1.56 (1.35-1.80)^***^

TTH, tension-type headache; IR, incidence density rates, per 1000 person-years; HR, hazard ratio; CI, confidence interval.

^†^ Patients with any one of diabetes, hyperlipidemia, hypertension, IHD, dementia, depression, migraine, stroke, CKD, and head injury were classified as the comorbidity group.

^‡^ Model mutually adjusted for sex, age, diabetes, hyperlipidemia, hypertension, IHD, dementia, depression, migraine, stroke, CKD, and head injury.

^*^ *P*<.05, ^**^ *P*<.01, ^***^ *P*<.001.

## DISCUSSION

To the best of our knowledge, this is the first nationwide, population-based longitudinal study to demonstrate a relationship between TTH and PD. Although the incidence of PD increased with age and comorbidities in both the TTH and non-TTH groups in our study, TTH was an independent risk factor for PD. The TTH group were 1.37-fold more likely to develop PD than the non-TTH group after adjustment for sex, age, and comorbidities. Notably, the higher risk of PD that was associated with TTH was not influenced by age, sex, or comorbidities after adjustment for multivariable effects.

The findings of the current study are inconsistent with those of previous clinic-based observational studies, which have demonstrated no significant difference [18] or a lower prevalence of headache [17, 19] in sex- and age-matched PD patients and controls. This discrepancy warrants careful consideration and interpretation. The observed differences between the results of our study and the findings of previous studies may be attributed to differences in the ethnicity of participants, environmental factors, methodology, and clinical settings. Moreover, our data were bolstered by several methodological and statistical strengths relative to previous studies. First, our study evaluated a comparatively broad age range in a large sample from the general population. Second, our study was carefully designed to account for possible confounding factors and comorbidities of PD that were potentially not addressed in previous studies. Third, in our results, the higher risk of PD in the TTH group was not influenced by the comorbidity status. This finding reinforces our conclusion that TTH is an independent risk factor for PD.

The possible comorbidities of PD, including diabetes, hyperlipidemia, hypertension, IHD, depression, stroke, head injury, and migraine, were more prevalent in the TTH group than in the non-TTH group in the present study. This is consistent with accumulating evidence that TTH may be comorbid with several medical and psychiatric conditions [21–23]. Nevertheless, although the TTH group exhibited a higher prevalence of the aforementioned comorbidities than the non-TTH group did, multivariate Cox analyses demonstrated that TTH was still an independent predictor of subsequent PD. In this study, the incidence of PD was higher in men than in women in both TTH and non-TTH groups (Table 3). This observation was consistent with the previous finding of a incidence of PD in men than in women [24]. However, regardless of the subject's sex, individuals in the TTH group had a higher risk for PD than those in the non-TTH group after adjustment for multiple covariates. This may further support the idea that TTH has an independent role in the development of PD.

In addition to examining the relationship between TTH and PD, our study investigated the other risk factors for PD. In our multivariate analysis, older patients and those with diabetes, hypertension, IHD, dementia, depression, or migraine had a significantly higher risk of PD after adjustment for covariates. These findings are consistent with previous reports that comorbidities such as diabetes, hypertension, IHD, dementia, and depression are risk factors for PD [2, 5, 25]. Moreover, the incidence of PD is higher in aging populations [2]. In the present study, the risk of PD was highest in those aged 45–64 and ≥65 years. However, in this study, no obvious sex-based differences were noted in PD occurrence (incidence) (Table 2). Our incidence results may conflict with those of studies carried out in Chinese or Western populations reported a higher incidence rate of PD in men than women [26, 27]. This discrepancy may have been due to environmental or genetic factors, but might have also been a consequence of differences in methodology. More comprehensive studies of the comparability of existing reports may be warranted in the future. Finally, our observation of higher adjusted HRs for PD in those with migraine supports the potential role of migraine as a risk factor for PD [6].

There are several potential mechanisms for the pathophysiological connection between TTH and PD. There are several potential mechanisms underlying the pathophysiological connection between TTH and PD. First, dopamine has been suggested to play a critical role in central pain modulation [28], and human neuroimaging studies have shown that pain processing involves dopamine D2 receptors [29]. Additionally, dopamine may modulate pain at different levels of pain processing, such as the spinal cord, thalamus, peri-aqueductal gray matter, basal ganglia, and cingulate gyrus [30]. Axons from important dopaminergic areas, including the substantia nigra pars compacta and the ventral tegmental area, project to the mesocortical, mesolimbic, and nigrostriatal pathways [30]. These pathways are associated with different non-motor symptoms in patients with PD, such as pain and cognition. Furthermore, patients with PD exhibit lower pain tolerance thresholds than healthy individuals. This may result from impaired descending inhibition of nociception from supraspinal structures [31]. Deficient descending pain inhibition has also been reported to play a role in the pathophysiology of chronic TTH [32]. Thus, reduced dopaminergic function and impaired descending pain inhibition from supraspinal sites are potential shared mechanisms between PD and TTH. Second, in addition to dopamine dysfunction, serotonergic dysfunction has been implicated in the pathophysiology of PD [33, 34]. Serotonergic cell loss has been reported to occur even before nigrostriatal dopaminergic degeneration in PD [35]. In fact, previous animal research indicates that serotonin secretion is blocked following the blockage of postsynaptic D2 receptors, and that serotonin secretion in the forebrain is dependent on intact local dopaminergic neurotransmission [36]. Serotonin has been reported to play an important role in pain processing. In addition, serotonergic dysfunction contributes to decreased descending inhibition of pain and the central sensitization of nociceptive pathways, which are thought to be associated with the pathophysiology of TTH [37]. Given the overlap between these processes, TTH may have a potential pathological association with PD. Additional studies should be carried out to explore the hypothesis that dopaminergic and serotonergic dysfunction underlie both TTH and PD.

Our present findings have several clinical implications. First, our results suggest that TTH may be viewed as a potential risk factor for PD. Due to the potential risk for subsequent PD, clinicians should consider screening for early non-motor features of PD in patients with TTH, particularly those who are elderly and those with medical comorbidities of PD. This might be one approach for the early preclinical diagnosis of PD. In addition, our finding that TTH increases the risk of PD independent of age and sex is notable given that most PD patients are older than 60 years [1]. Furthermore, PD is usually more common in men than in women [24]. We therefore believe that the influence of age or sex on the TTH–PD relationship warrants further study. Pain and depression are two common and distressing nonmotor symptoms in patients with PD [9, 38]. Cumulative evidence suggests an association between pain and depression in PD, as both pain and depression are important contributors to reduced quality of life in patients with PD [39–41]. TTH is one of the common causes of pain. Taken together our results, this suggest that understanding the relationship between pain, including TTH, and depression in patients with PD may improve future treatment strategies and quality of life in patients with PD.

The major strengths of the present study are the use of nationwide population-based data and the longitudinal, observational design with a long follow-up period. A large sample size provided sufficient statistical power to explore the potential relationship between TTH and PD while various covariates were considered. Furthermore, in this study, the diagnosis of TTH was based on ICD-9 codes and was made by qualified clinical physicians as a part of a strictly audited reimbursement process. TTH diagnoses were based on the *International Classification of Headache Disorders, Second Edition* (ICHD-II) criteria [42], as the ICHD-III-beta [43] was not available until 2013, which was after the study was carried out. The NHIRD covers a highly representative sample of Taiwan's general population because the reimbursement policy is universal and operated by a single buyer, namely the Government of Taiwan. All insurance claims were required to be scrutinized and coded by medical reimbursement specialists and peer reviewed according to the criteria for diagnosis of TTH in this study. If doctors or hospitals committed errors in diagnoses or coding, they were punished with significant penalties. Indeed, the reliability and validity of the NHI research database for epidemiologic investigations have been reported [44]. Therefore, the diagnosis and coding of TTH in this study were highly reliable. Finally, our longitudinal study afforded an assessment of the temporal relationship between TTH and PD, which reveals the potential direction of the association between these two disorders.

Nevertheless, our study had some limitations. First, TTH and PD diagnoses were based on diagnostic codes that were entered by physicians into the NHIRD, and this database does not include additional information on TTH or PD, such as the duration, severity, and frequency of TTH or the stage and prognosis of PD. Thus, we could not differentiate between episodic or chronic TTH patients or determine the relationship of the PD stage or prognosis with TTH headache characteristics. Second, the NHIRD does not provide detailed information on patients’ lifestyle habits, such as alcohol consumption, smoking habits, genetic factors, family history, and other environmental factors. Accordingly, these factors could not be controlled for in our analysis and may have exerted confounding effects. Finally, we identified a temporal association between TTH and PD by using epidemiologic data from a large healthcare database; investigations of the causal relationship and underlying mechanisms between these two disorders are still warranted.

In conclusion, we used a large-scale, nationwide, population-based, longitudinal study to validate a temporal association between TTH and PD. Although the precise underlying mechanisms linking TTH and PD remain unclear, our findings provide insight into the possible shared pathophysiology between TTH and PD. Future studies should examine the mechanism and causality of this clinically critical association.

## MATERIALS AND METHODS

### Data source

The National Health Insurance (NHI) program of Taiwan, implemented in March 1995, is a mandatory single-payer healthcare system, and by 2014, it covered approximately 99.9% of Taiwanese residents. The National Health Insurance Research Database (NHIRD) is maintained by the National Health Research Institutes (NHRI), Taiwan, and is accessible to scientists for research purposes. In this study, we used the data sets of the Registry for Longitudinal Health Insurance Database 2000 (LHID2000), which is a subset of the NHIRD. The LHID2000 contains all the claims data of 1 million randomly sampled beneficiaries who were enrolled in the NHI program from 1996 to 2000. All the records of these individuals from 1996 to 2011 were collected. The NHRI reported no statistically significant differences in the sex and age distribution between the LHID2000 and all the beneficiaries of the NHI program. The LHID2000 contains demographic data, inpatient and outpatient information, the date of visit or hospitalization, drug prescriptions, and diagnoses coded in the format of the International Classification of Disease, Ninth Revision, Clinical Modification (ICD-9-CM).

### Ethics statement

The NHIRD encrypts patient personal information to protect privacy and provides researchers with anonymous identification numbers associated with relevant claims information, including sex, date of birth, medical services received, and prescriptions. Therefore, patient consent is not required to access the NHIRD. This study was approved to fulfill the condition for exemption by the Institutional Review Board (IRB) of China Medical University (CMUH-104-REC2-115). The IRB also specifically waived the consent requirement.

### Study population

We conducted a retrospective population-based cohort study that included two groups: the TTH and non-TTH groups. We identified 13,384 patients who were newly diagnosed with TTH (ICD-9-CM codes 307.81 and 339.1) from 2000 to 2005, who were included in the TTH group. TTH diagnoses were based on the *International Classification of Headache Disorders, Second Edition* (ICHD-II) criteria [42]. The index date was defined as the date of the first diagnosis of TTH. To ensure the accuracy of the TTH diagnostic code, we selected patients with at least two outpatient visits or at least one inpatient hospitalization. We excluded patients with missing sex or age information (*n* = 2), those aged younger than 20 years (*n* = 875), and those diagnosed with PD (ICD-9-CM code 332) before the index date (*n* = 198) from the analyses. For each TTH patient, four insurants without TTH were randomly selected from the LHID2000 for inclusion in the non-TTH group and were frequency matched by sex, age (every 5 years), and the index date by using the same inclusion criteria that were used for those in the TTH group. Finally, 12,309 patients were included in the TTH group, and 49,236 subjects were included in the non-TTH group.

Demographic factors included sex and age (20–44, 45–64, and ≥65 age groups). Although PD mostly affects older individuals aged 65 years or older, it may occur in individuals aged between 45 and 64 years of age, and even in younger individuals, in which case it is known as young-onset PD [45]. The average age of onset for TTH is 25 to 30 years, and the peak prevalence of TTH occurs at ages between 30 and 39 years [46]. Therefore, we considered age to be a three-level ordinal variable (with cutoffs at 45 and 65 years). From the claims data, we also recorded comorbidities occurring before the index date, including diabetes (ICD-9-CM code 250), hyperlipidemia (ICD-9-CM code 272), hypertension (ICD-9-CM codes 401–405), ischemic heart disease (IHD; ICD-9-CM codes 410–414), dementia (ICD-9-CM codes 290, 294.1, 331.0), depression (ICD-9-CM codes 296.2, 296.3, 300.4, and 311), migraine (ICD-9-CM code 346), stroke (ICD-9-CM codes 430–438), chronic kidney disease (ICD-9-CM code 585), and head injury (ICD-9-CM codes 310.2, 800, 801, 803, 804, 850–854, and 959.01).

In this study, the endpoint of interest was PD occurrence (ICD-9-CM code 332). Both groups were followed up from the index date to the date of PD diagnosis, withdrawal from the NHI program, or the end of 2011.

### Statistical analyses

The descriptive statistics of the two groups are presented as a number and percentage for categorical variables and as a mean and standard deviation (SD) for continuous variables. We compared the distributions of demographic factors and comorbidities between the groups through the Pearson's chi-square test for categorical variables and the Student's t test for continuous variables. The incidence density rates were calculated by dividing the number of PD patients by the number of person-years. We estimated the cumulative incidence curves of PD for both groups through the Kaplan–Meier method and assessed their differences through the log-rank test. We used Cox proportional hazards regression to calculate the hazard ratios (HRs) and 95% confidence intervals (CIs) of PD to evaluate the independent effect of TTH after adjusting for sex, age, and comorbidities.

All statistical analyses were performed using SAS, version 9.4 (SAS Institute, Cary, NC, USA). Two-sided *P*<.05 were considered statistically significant.

## Abbreviations

CIs: confidence intervals
HRs: hazard ratios
ICD-9-CM: International Classification of Disease
IHD: ischemic heart disease
IRB: Institutional Review Board
LHID2000: Longitudinal Health Insurance Database 2000
NHI: National Health Insurance
NHIRD: National Health Insurance Research Database
NHRI: National Health Research Institutes
PD: Parkinson's disease
SD: standard deviation
TTH: tension-type headache
